# Effects of red and blue light on growth and photosynthesis of *Populus szechuanica* var. *tibetica*

**DOI:** 10.3389/fpls.2025.1712729

**Published:** 2025-11-24

**Authors:** Rong Xu, Shihai Zhang, Cai Wang, Xiaolin Zhang, Lihong Zhao, Dan Zong

**Affiliations:** 1 Forest Resources Exploitation and Utilization Engineering Research Center for Grand Health of Yunnan Provincial Universities, Southwest Forestry University, Kunming, China; 2 College of Biological Science and Food Engineering, Southwest Forestry University, Kunming, China; 3 College of Forestry, Southwest Forestry University, Kunming, China

**Keywords:** *Populus szechuanica*, light quality, blue light, photosynthesis, rubisco

## Abstract

**Introduction:**

*Populus szechuanica* var. *tibetica* is a high-altitude tree species. At elevated altitudes, the proportion of short-wave light increases, suggesting that *P. szechuanica* var. *tibetica* has likely evolved unique adaptive mechanisms to withstand such conditions.

**Methods:**

In this study, one-year-old *P. szechuanica* var. *tibetica* branches were cultivated in seedling bags and subjected to different light treatments: white light (W control), monochromatic red light (R), and mixed red-blue light (B) systems with varying ratios (R1B3, R3B1), to investigate the effects of red-blue light quality on *P. szechuanica* var. *tibetica* morphology, leaf anatomical structure, photosynthetic performance, and photosynthetic metabolism.

**Results:**

The results demonstrated that monochromatic red light enhanced *P. szechuanica* var. *tibetica* growth and leaf expansion while reducing photosynthetic efficiency. In contrast, mixed red-blue light treatments - particularly the R1B3 ratio - induced a compact growth morphology with thicker, smaller leaves. These structural modifications corresponded with improved adaptation to high-altitude environments where short-wave light constitutes a greater proportion of the solar spectrum. In terms of photosynthetic characteristics, R1B3 enhances photosynthesis in *P. szechuanica* var. *tibetica* through a synergistic effect of increasing the number of stomata on the upper epidermis of leaves and improving stomatal conductance, as well as significantly increasing Rubisco activated enzyme (RCA) content and Rubisco activity. Collectively, *P. szechuanica* var. *tibetica* under R1B3 light treatment adapt to altered light quality by modifying their growth morphology, leaf anatomy, and photosynthetic metabolites, resulting in enhanced photosynthetic efficiency.

**Discussion:**

This study provides theoretical support for the adaptation mechanism of *P. szechuanica* var. *tibetica* to different light qualities, but the ratio of red to blue light qualities needs further refinement. In subsequent research, the ratio can be refined and the proportion of blue light increased to deepen the study.

## 1 Introduction

Plants are photographically organisms whose entire life cycle depends on light. Light is one of the most critical environmental factors influencing plant growth and development, as it not only serves as the ultimate energy source for metabolism in green plants but also functions as a signal that activates and regulates processes associated with photomorphogenesis (Wei and Yu, 2023). Light influences the photosynthetic characteristics of seedlings by regulating chloroplast development, modulating the activity of key enzymes, and affecting gene expression associated with the Calvin cycle, among other processes (Kreslavski et al., 2013; Gutu et al., 2013; Albrecht-Borth et al., 2013).

Light signals-encompassing light intensity, light period and spectral distribution-as key components modulate nearly all stages of plant growth and development across their entire life cycle (Jiao et al., 2007). Spectral distributions, especially red (R, 650 nm) and blue (B, 450 nm) wavelengths, are involved in plant photosynthesis by providing energy required for plant growth and development and regulate seedling photomorphogenesis, stomatal aperture, chlorophyll content, photosynthesis, and gene expression (Tarakanov et al., 2021, 2022; Li and Liu, 2021; Yang et al., 2018; Zheng and Van Labeke, 2017). Previous studies have shown that blue light regulates seedling growth, leaf expansion, and chloroplast development (Liu et al., 2013; Hernández and Kubota, 2016) and it also plays a role in stomatal regulation, affecting gas exchange and water loss (Shimazaki et al., 2007). Conversely, red light has been shown to significantly promote photosynthetic activity, plant biomass accumulation, and leaf area expansion, while also playing a critical role in the development of the photosynthetic apparatus, enhancement of net photosynthetic rate (*Pn*), and regulation of primary metabolism (Hernández and Kubota, 2016; Demotes-Mainard et al., 2016). Related studies showed that blue light increases leaf photosynthetic pigment content and improves the stomatal conductance (Hogewoning et al., 2010; Johkan et al., 2010) and the leaf area decreased as the blue photon flux increased, when cucumber seedlings were exposed to a combination of R and B light (Hernández and Kubota, 2016). It has been suggested that red light alone was unsuccessful for chlorophyll biosynthesis, whereas the combination of blue and red light was necessary for this process (Fan et al., 2013). The combination of blue and red light has significant effects on plant growth, and the ratio of blue to red light profoundly influences plant morphology, pigmentation and overall growth patterns (Seyedi et al., 2024). Given that each plant has specific light requirements, Understanding the precise light preferences of the target plant is crucial for optimizing its morphology, pigmentation, and overall growth patterns (Li and Kubota, 2009).

*Populus szechuanica* var. *tibetica* belongs to *Populus* sect. *Tacamahaca* in the genus *Populus* and is an ecologically important species, mainly distributed in Sichuan and Tibet at altitudes from 2000 to 4500 m (Chen et al., 1994). Generally speaking, as the altitude gradient increases, the light intensity rises and the short-wave radiation strengthens (Pan et al., 2009). Given that different light wavelengths exert varying degrees of influence on plant processes such as photosynthesis, pigment formation, and morphogenesis, plants at different altitudes can adapt to the light environment through morphological changes (Wang, 1989; Castro et al., 2000). Recent studies reported that the growth of *P. szechuanica* var. *tibetica* was superior to other species, when eight *Populus* species from different altitudes in Southwest China were introduced to Kunming (Zong and Wang, 2022). Comparing the spectra of low-altitude regions (Beijing) and high-altitude regions (Lhasa), it was found that blue light levels in high-altitude areas are 20% higher than in low-altitude areas (Tunzhup et al., 2022; Liu et al., 2000). Furthermore, when *P. szechuanica* var. *tibetica* was treated with blue film, it can increase the photosynthetic pigment content, improve the utilization efficiency, and enhance photosynthetic efficiency, and promote the accumulation of photosynthesis (Liu et al., 2024). Blue light film treatment of another high-altitude poplar species, *Populus schneideri*, also significantly enhanced its photosynthetic characteristics and growth development (Chen et al., 2024). In terms of the spectral properties of the films, two other measurements of the transmitted spectral components of blue and white films showed that the red-to-blue light ratios under blue film and white film were 1:3 and 3:1, respectively. Therefore, to better understand the effect of light quality on plant growth and photosunthetic performance of *P. szechuanica* var. *tibetica*, White (W), Red (R), Red: Blue=3:1 (R3B1) and Red: Blue=1:3 (R1B3) spectra were used to investigate their impact on growth metrics, leaf morphological indices, photosynthetic parameters and chlorophyll content. The results will provide some basic information for subsequent research on the adaptation mechanisms of forest trees to different light qualities.

## 2 Materials and methods

### 2.1 Plant materials and experimental setup

**Figure 1:**
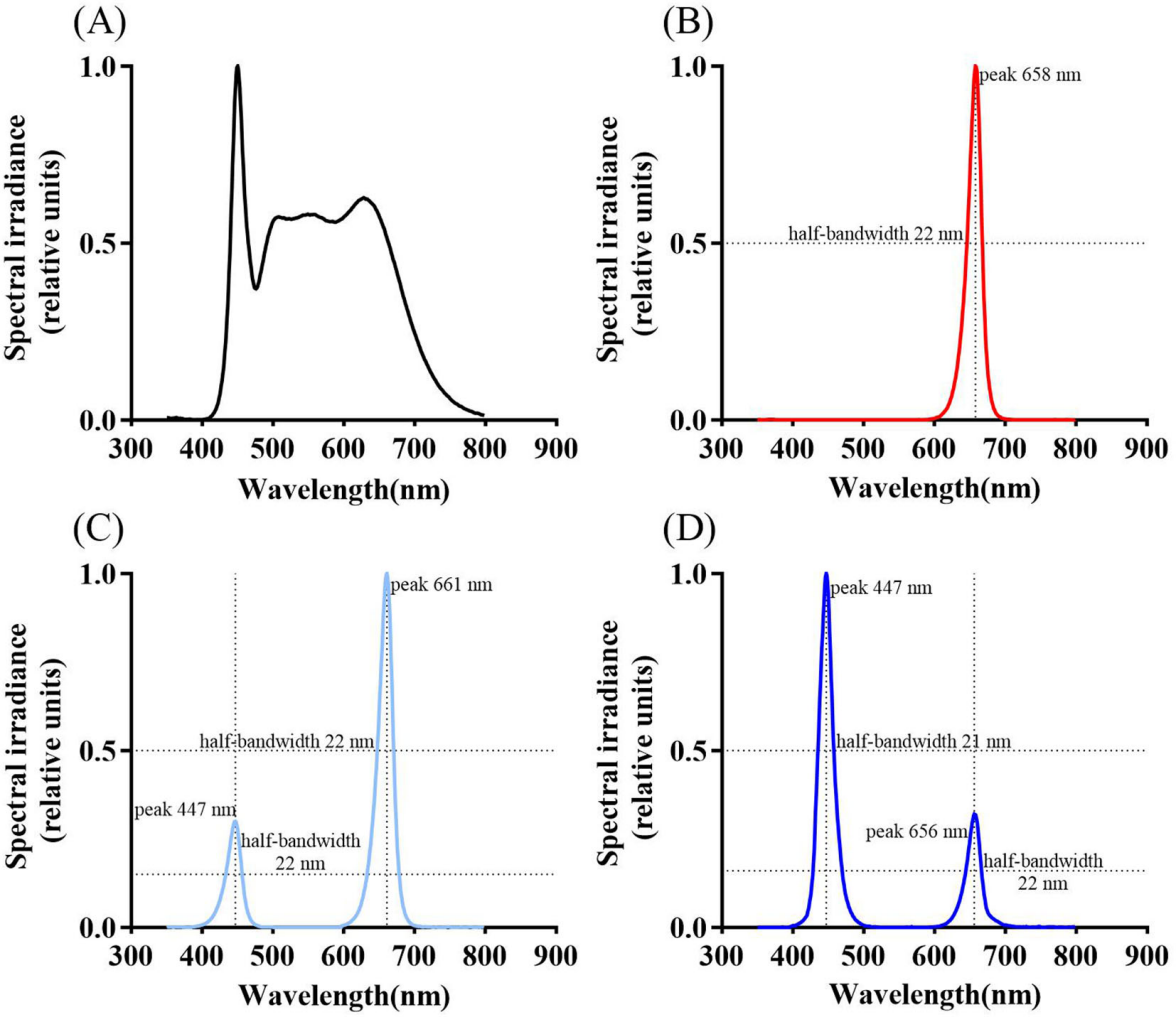
Spectral diagrams under different treatments: CK [W, **(A)**], red light treatment [R, **(B)**], the ratio of photosynthetic photon flux density (PPFD) between red light and blue light is 3:1 [R3B1, **(C)**], the ratio of photosynthetic photon flux density (PPFD) between red light and blue light is 3:1 [R1B3, **(D)**]. Peak wavelengths and half-bandwidths for each treatment spectrum are labeled in the figure.

*Populus szechuanica* var. *tibetica* specimens were collected from Markam County, Tibet Autonomous Region (98°13′00″E, 29°32′36″N; altitude 3533 m). These plants were propagated via hardwood cuttings and transplanted to a poplar common garden located in Aziying, Kunming for cultivation. This study selected healthy one-year-old branches of *P szechuanica* var. *tibetica* growing in Azying, Kunming. Cuttings 15 cm in length were taken, each bearing two lateral buds. After soaking in water for 24 hours, the cuttings were transplanted into 15 cm diameter seedling bags. The culture medium consisted of field red soil, biochar, and humus mixed in a 1:2:1 volume ratio. Each seedling bag contained two cuttings (144 bags total) and was placed in a constant-temperature incubator under white light for 40 days (temperature 25°C, relative humidity 70%, light intensity 100 μmol m^-^² s^-^¹, under a 12-hour light/12-hour dark photoperiod). After 40 days, uniformly growing cuttings (72 bags total) were evenly distributed among four light quality treatments: white light (W, serving as the control), red light (R), the ratio of photosynthetic photon flux density (PPFD) between red light and blue light is 3:1 (R3B1) and 1:3 (R1B3) for 60 days (temperature 25°C, relative humidity 70%, light intensity 100 μmol m^-^² s^-^¹, 12-hour light/dark cycle). Subsequent experiments were conducted on these seedlings. All samples (144 bags and 72 bags) comprised three clones, with each clone representing one biological replicate, and each biological replicate contained an equal number of samples. Following an additional 60 days of cultivation under a 12-h light/12-h dark photoperiod, physiological parameters were determined. The LED light sources used (F-grade; 5000K; wavelength range 380–780 nm) were purchased from Ningbo Jiangnan Instrument Factory. Spectral diagrams of different treatments are shown in **Figure 1**.

### 2.2 Measurement of morphological and physiological indicators

#### 2.2.1 Measurement of growth indicators

Plant height was defined as the length of the primary shoot, and basal diameter was measured at 2 cm above the root-shoot junction, with a measuring tape and a vernier caliper used for these measurements, respectively. For each biological replicate, three plants were randomly selected from 12 cuttings, tagged, and their initial height and basal diameter were measured and recorded. After 60 days of treatment, the tagged plants were re-measured for height and basal diameter, and absolute growth increments were calculated as the difference between the final and initial measurements. All data were subsequently subjected to statistical analysis.

#### 2.2.2 Determination of leaf shape index and anatomical structure

After 60 days of cultivation under red and blue light, select the 5 to 7 leaves growing below the main branch apex of *P. szechuanica* var. *tibetica* under light quality treatment. Use the YMJ-C leaf area meter to determine leaf shape indices (including leaf area, perimeter, length, and width). For each clone, three plants were measured, resulting in a total of nine (Zhou and Zhang, 2018) plants per treatment. The methodology for leaf anatomical observations followed that of Zhou Naifu (Zhou and Zhang, 2018). For microscopic examination, 2 cm × 2 cm tissue segments were excised from the 5th leaf (counted from the apex) of the selected plants. One plant was sampled per clone, resulting in a total of three plants per treatment. These tissue segments were sectioned to a 4 μm thickness using a Leica RM2016 microtome, then subjected to deparaffinization and stained with Safranin-Fast Green Staining. Subsequently, the sections were observed under a NIKON ECLIPSE C1 upright microscope, which was equipped with a NIKON Digital Sight DS-F12 imaging system.

We further employed ImageJ software to determine parameters related to leaf tissue structure, including leaf thickness (LT), palisade mesophyll thickness (PT), spongy mesophyll thickness (ST), upper epidermal thickness (UE), and lower epidermal thickness (LE). For each sample, ten fields of view were analyzed, and the mean values obtained were used to calculate the leaf cell tense ratio (CTR), leaf spongy ratio (SR), and the PT/ST ratio using the following formulae:


$$CTR=(\frac{PT}{LT})\times 100\%$$

$$SR=(\frac{ST}{LT})\times 100\%$$

#### 2.2.3 Measurement of photosynthetic parameters

After 60 days of treatment, photosynthetic gas exchange parameters were measured using an LI-6800 portable photosynthesis system (LI-COR, USA) to evaluate the effects of red and blue light on the leaves of *P. szechuanica* var. *tibetica* under different light treatments. All measurements were conducted between 9:00 and 11:00 on clear mornings under incubator light conditions. For each measurement, the 5th to 7th leaves from the top of the main branch were selected as experimental materials. The parameters determined included net photosynthetic rate (*P_n_*), stomatal conductance (*G_s_*), transpiration rate (*T_r_*), and intercellular CO_2_ concentration (*C_i_*). Three biological replicates were set for each asexual line in the measurements.

After 60 days of treatment, light response and CO_2_ response curves were measured on the fifth leaf from the top of the main branch using an LI-6800 portable photosynthesis system (LI-COR, USA). All measurements were conducted between 8:00 and 11:00 on clear mornings. For the light response curve measurements: the leaf chamber CO_2_ concentration was maintained at 400 µmolmol^-1^, while the photosynthetically active radiation (PAR) was set in decremental steps from 2000 to 0 µmolm^-2^s^-1^ using the instrument’s built-in red and blue light source (Duan and Yan, 2019). For the CO_2_ response curve measurements: PAR was fixed at 1800 µmolm^-2^s^-1^, and the CO_2_ concentration gradients were set as follows: 400, 300, 200, 100, 50, 20, 10, 400, 400, 600, 800, 1000, 1200, 1400, 1600, 1800, 2000, 2200 µmolmol^-1^ (Duan and Yan, 2019). Three biological replicates were included for each asexual line (one plant per line).

#### 2.2.4 Determination of photosynthetic pigments and products

After 60 days of treatment, photosynthetic pigments and products were analyzed. For each treatment, samples were collected from three asexual lines, with three plants sampled per line. From each plant, the 5th to 7th leaves counting from the apex downward on the main branch were collected, the main midrib was removed, and the material from all three plants within an asexual line was chopped, mixed, and pooled to form a single composite sample per line. For pigment quantification, photosynthetic pigments were extracted using the ethanol grinding extraction method, and absorbance was measured at 665 nm (A_665_), 649 nm (A_649_), and 470 nm (A_470_) using a visible spectrophotometer. Each composite sample was measured three times. Chlorophyll a (Chl a), chlorophyll b (Chl b), and carotenoid (Car) contents were calculated according to Equation (Cai, 2014).

Total soluble sugars, reducing sugars, and starch were extracted using the anthrone-sulfuric acid and 3,5-dinitrosalicylic acid (DNS) methods, respectively. Absorbance was measured with a visible spectrophotometer, and sucrose/starch contents were calculated using their respective standard curves according to Reference (Gao, 2006).

#### 2.2.5 Determination of photosynthetic enzyme content and activity

In this experiment, both the determination of photosynthetic enzyme (Rubisco activating enzyme (RCA), ribulose-1,5-bisphosphate carboxylase (Rubisco), and sedoheptulose-1,7-bisphosphatase (SBPase)) content and activity were performed using the enzyme-linked immunodeficient assay (ELISA) method, employing the ELISA kit provided by Beijing Chenglin Biotechnology Co., Ltd.

For each biological replicate, take 0.1 g of leaves (the 5th to 7th leaves counting down from the leaf tip) with the main veins removed and store them frozen in liquid nitrogen. Place a liquid nitrogen-precooked mortar on ice. Add 1 mL of PBS buffer (pH 7.4), then add the leaves and homogenize in an ice bath. Transfer the homogeneity to an EP tube and centrifuge at 8000 rpm for 15 minutes. After collecting the supernatant, determine the enzyme content and activity according to the instruction manual.

All test solutions for enzyme content and activity were prepared separately using the aforementioned method.

#### 2.2.6 Statistics and analysis

The measured data were analyzed using Excel 2019 and SPSS 21.0. Differences among treatments were evaluated by one-way analysis of variance (ANOVA), followed by Duncan’s multiple range test for *post-hoc* comparisons, with statistical significance set at P < 0.05. Figures were generated using GraphPad Prism 8.0.

The non-rectangular hyperbolic model and rectangular hyperbolic model were fitted to the photosynthetic light response (*P_n_*-PAR) curves and CO_2_ response (*P_n_*-CO_2_) curves, respectively (Lewis and Olszyk, 1999; Thornley, 1976; Baly, 1935).

## 3 Results

### 3.1 Impact of different light treatments on growth

**Figure 2:**
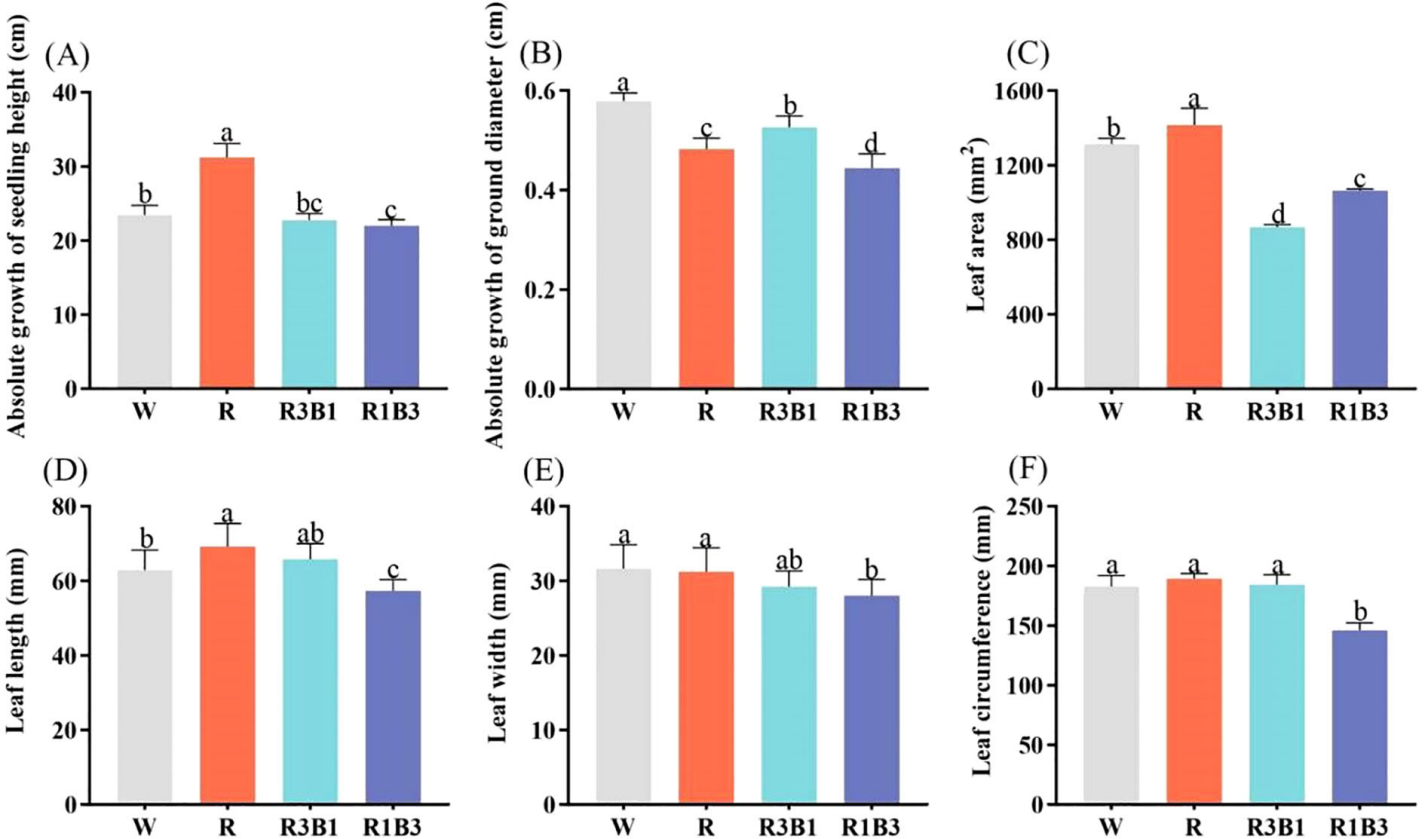
Effects of light quality on the growth of *P. szechuanica* var. *tibetica* heights **(A)**, ground diameter **(B)**, leaf area **(C)**, leaf length **(D)**, leaf width **(E)**, and leaf circumference **(F)**. Different lowercase letters indicate significant differences among treatments at *P* < 0.05. White: W, Red: R, R3B1 and R1B3: The ratio of photosynthetic photon flux density (PPFD) between red light and blue light is 3:1and 1:3.

Light quality significantly affected the growth of *P. szechuanica* var. *tibetica* seedlings (**Figure 2**). The highest absolute growth of seedling height was in the red light (R) treatment, the plant height in this treatment was significantly higher than that in the CK (W, increase height by 25%), R3B1 (increase height by 27%) and R1B3 (increase height by 30%) treatments (**Figure 2A**). As the ratio of blue light (B) increased, the absolute growth of seedling height gradually decreased. The smallest absolute growth of ground diameter was in the R1B3 treatment, the ground diameter in this treatment was significantly lower than that in the CK (W, reduced by 24%), R (reduced by 8.3%) and R3B1 (reduced by 15.4%) treatments (**Figure 2B**).

**Figure 3:**
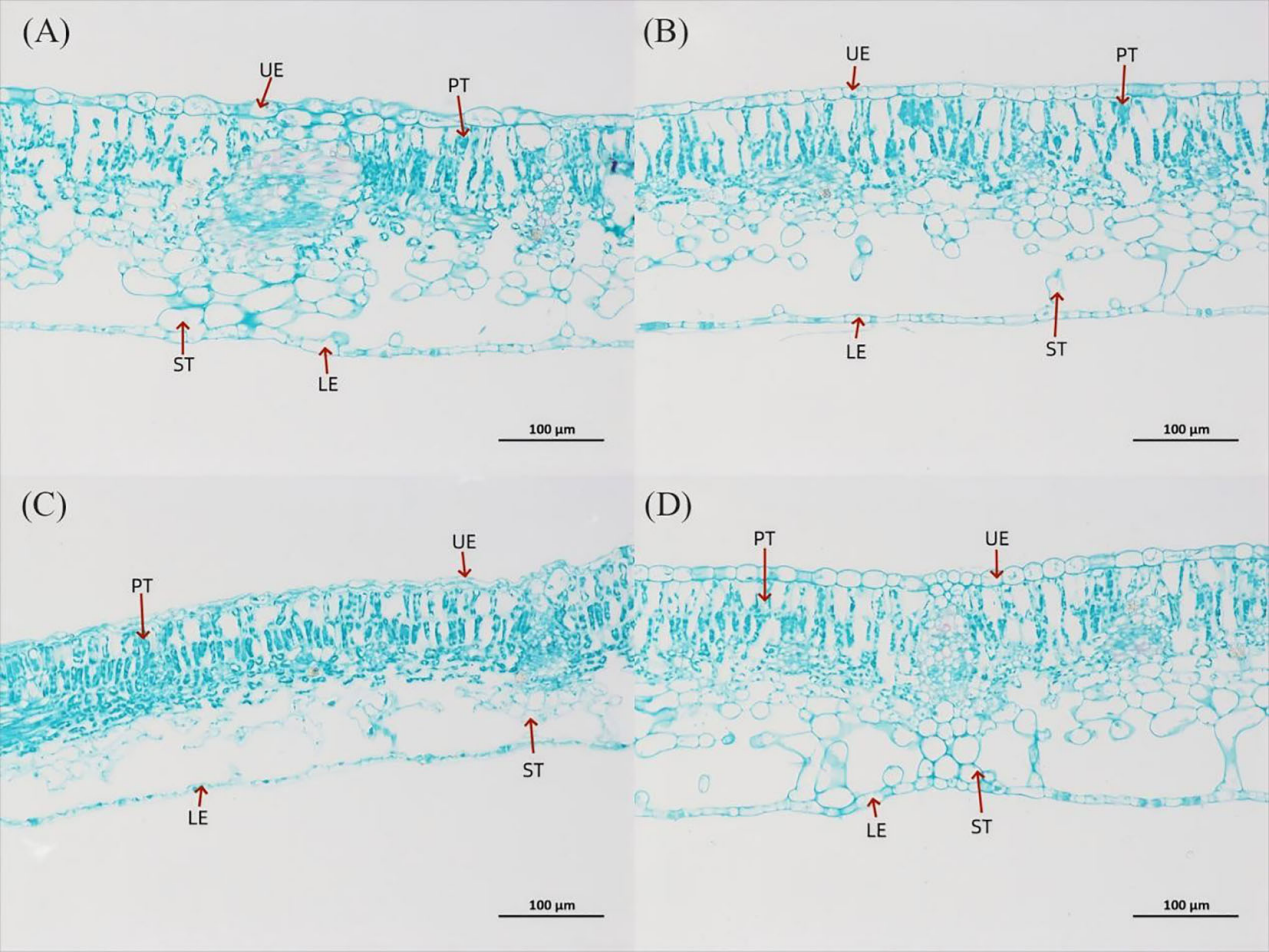
Leaf anatomical structure of *P. szechuanica* var. *tibetica* under different light quality treatments (saffron solid green staining), magnification 200×. **(A)** W treatment, **(B)** R treatment, **(C)** R3B1 treatment, and **(D)** R1B3 treatment. The figure in the table represents: upper epidermis thickness (UE), lower epidermis thickness (LE), palisade tissue (PT) and spongy tissue thickness (ST).

As shown in **Figure 3**, the leaf characteristics of *P. szechuanica* var. *tibetica* seedlings were significantly influenced by light qualities. Leaf area, leaf length, and leaf circumference were highest under R treatment (**Figures 2C, D, F**). Leaf width, leaf length, and leaf circumference were lowest under R1B3 treatment (**Figures 2D–F**). Compared to the R treatment, leaf area, leaf length, and leaf width decreased by 25%, 17%, and 23%, respectively, under the R1B3 treatment.

### 3.2 Impact of different light treatments on leaf anatomical structure

The internal structure of *P. szechuanica* var. *tibetica* seedling leaves changed under different light quality treatments (**Figure 3** and **Tables 1**, **2**). The results indicated that red light inhibited the number of stomata in both the upper (reduce 29%) and lower (reduce 36%) epidermises of *P. szechuanica* var. *tibetica* seedlings. In contrast, the R3B1 and R1B3 treatments increased stomatal counts in the upper epidermis while suppressing those in the lower epidermis (**Table 1**).

**Table 1:** Effect of light quality on the number of leaf stomata.

Treatment	Stomatal number on upper epidermis (/mm²)	Stomatal number on lower epidermis (/mm²)
W	158.02 ± 18.32^c^	1439.51 ± 94.69^a^
R	122.63 ± 25.81^d^	919.34 ± 75.96^d^
R3B1	209.05 ± 22.08^b^	1259.26 ± 109.69^b^
R1B3	274.9 ± 41.97^a^	1004.94 ± 62.49^c^

Different lowercase letters indicate significant differences among treatments at *P* < 0.05. White: W, Red: R, R3B1 and R1B3: The ratio of photosynthetic photon flux density (PPFD) between red light and blue light is 3:1and 1:3.

**Table 2:** Comparison of the anatomical structure of the leaves of *P. szechuanica* var. *tibetica* under different treatments.

Treatment	LT/μm	UE/μm	LE/μm	PT/μm	ST/μm	CTR/%	SR/%	PT/ST
W	206.08 ± 10.83^b^	12.61 ± 0.63^b^	9.74 ± 0.82^a^	55.75 ± 3.59^b^	132.15 ± 8.00^b^	27 ± 0.01^b^	64 ± 0.04^a^	0.42 ± 0.03^bc^
R	229.44 ± 3.90^a^	13.34 ± 0.90^a^	8.39 ± 0.62^b^	58.65 ± 2.39^a^	146.86 ± 5.59^a^	26 ± 0.01^c^	64 ± 0.02^a^	0.40 ± 0.03^c^
R3B1	196.92 ± 3.51^c^	9.3 ± 1.30^c^	7.92 ± 0.97^b^	53.22 ± 1.42^c^	124.29 ± 3.15^c^	27 ± 0.01^b^	63 ± 0.01^a^	0.43 ± 0.01^b^
R1B3	205.43 ± 8.15^b^	12.01 ± 1.05^b^	9.20 ± 0.88^a^	59.17 ± 1.89^a^	115.79 ± 4.24^d^	29 ± 0.02^a^	56 ± 0.03^b^	0.51 ± 0.02^a^

Different lowercase letters indicate significant differences among treatments at *P* < 0.05. The letters in the table represent: leaf thickness (LT), upper epidermis thickness (UE), lower epidermis thickness (LE), palisade tissue thickness (PT), spongy tissue thickness (ST), leaf cell tense ratio (CTR), leaf sponge ratio (SR) and PT/ST. White: W, Red: R, R3B1 and R1B3: The ratio of photosynthetic photon flux density (PPFD) between red light and blue light is 3:1and 1:3.

**Figure 4:**
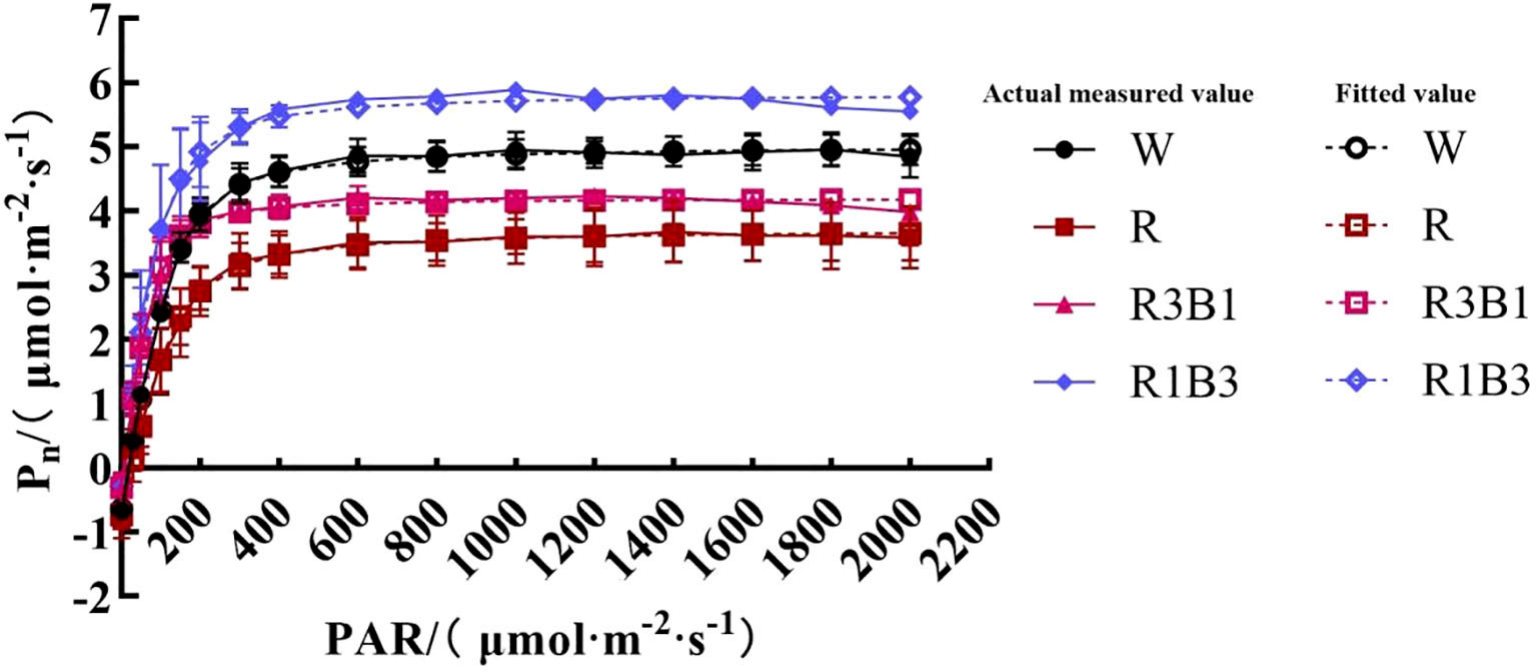
The response curves of *P_n_*-PAR of *P.szechuanica* var. *tibetica* under different treatments. Of which the dashed lines represent fitted values, and the solid lines represent measured values. White: W, Red: R, R3B1 and R1B3: The ratio of photosynthetic photon flux density (PPFD) between red light and blue light is 3:1and 1:3.

Under R treatment, the leaf thickness (LT, increase 10%), upper epidermis (UE, increase 5%), palisade tissue (PT, increase 5%), and spongy tissue (ST, increase 10%) were significantly increased, while the lower epidermis (LE, reduce 14%) and cell tension ratio (CTR, reduce 4%) were significantly decreased, when compared to W treatment (**Figures 3A, B**, **Table 2**). However, the LT, UE, LE, PT, and ST were significantly decreased under R3B1 treatment (**Figures 4A, C**, **Table 2**). Under R1B3 treatment, PT (increase 6%) and the ratio of PT/ST (increase 18%) showed significant increases, whereas the spongy tissue ratio (SR) was markedly reduced (**Figure 3D**).

### 3.3 Impact of different light treatments on photosynthetic parameters

The photosynthetic characteristics of *P. szechuanica* var. *tibetica* varied among light treatments (**Table 3**). The transpiration rate (*T_r_*) were significantly decreased by 36.77%, 36.77%, and 9.42%, respectively, (P<0.05) at R, R3B1, R1B3 treatment compared to W treatment. However, the net photosynthetic rate (*P_n_*) at R3B1, R1B3 treatment increased by 41.20% and 67.80%, respectively, (P<0.05) compared to W treatment. The intercellular CO_2_ concentration (*C_i_*) decreased significantly under different light quality treatments compared with the W treatment. Moreover, the photosynthetic characteristics of Tr, and stomatal conductance (*G_s_*) did not differ significantly under R and R3B1 treatment.

**Table 3:** Comparison of photosynthetic gas exchange parameters of *P. szechuanica* var. *tibetica* under different light quality.

Treatment	*T_r_ (mmol m^-^² s^-^¹)*	*P_n_ (µmol m^-^² s^-^¹)*	*C_i_ (µmol mol^-^¹)*	*Gs (mol m^-^² s^-^¹)*
W	2.23 ± 0.03^a^	1.77 ± 0.04^b^	361.49 ± 1.79^a^	0.11 ± 0.01^a^
R	1.41 ± 0.03^c^	1.73 ± 0.01^b^	346.12 ± 5.49^b^	0.07 ± 0.01^b^
R3B1	1.41 ± 0.01^c^	3.01 ± 0.16^a^	314.98 ± 3.69^d^	0.07 ± 0.01^b^
R1B3	2.02 ± 0.11^b^	2.97 ± 0.09^a^	335.71 ± 3.02^c^	0.11 ± 0.02^a^

Different lowercase letters indicate significant differences among treatments at *P* < 0.05. The same as below. The letters in the table represent: Transpiration rate (*T_r_*), Stomatal conductance (*G_s_*), and net photosynthetic rate (*P_n_*), Intercellular CO_2_ concentration (*C_i_*). White: W, Red: R, R3B1 and R1B3: The ratio of photosynthetic photon flux density (PPFD) between red light and blue light is 3:1and 1:3.

The response of *P_n_* to PAR was significantly diverse under different light quality treatments (**Figure 4**). The *P_n_*-PAR response curves could be divided into three stages, among which the first and second stage showed a similar trend of the response. In the first stage, where PAR <200 µmol m-² s-¹, *P_n_* increased linearly as PAR increased. With the further increases of PAR, the curves entered the second stage, *P_n_* increased curvilinearly to saturation, and *P_nmax_* appeared. The *P_n_*-PAR response curves in the third stage were significantly different under different light quality treatments. However, under R1B3 and R3B1 treatments, *P_n_* reached saturation at PAR of 1600 µmol m-² s-¹, while W and R appeared at 1800 µmol m-² s-¹, then *P_n_* decreased slowly and mild photoinhibition was observed (**Figure 4**).

The *P_n_*-PAR response curves were well fitted by the modified rectangular hyperbolic model as indicated by *R*^2^ values, which were greater than 0.994 (**Table 4**). Under different light quality treatments, there were no significant differences in *AQE*. *R_d_* of the R treatment were significantly higher than those of the CK under W treatment, while under R3B1 and R1B3 treatments, R_d_ were significantly lower than those of the CK. Under all light quality treatments, the values of *LP*_nmax_ were significantly higher in the R1B3 than in the R3B1 and R treatments. The *LCP* values in R were significantly higher than those of the W; in contrast, the *LCP* values in R3B1 and R1B3 were significantly lower than those in W (**Table 4**).

**Table 4:** Comparison of *P*_n-_*PAR* response parameters of *P. szechuanica* var. *tibetica* under different light quality.

Treatment	*AQE* (*μmol m^-2^ s^-1^*)	*R_d_*(*μmol m^-2^ s^-1^*)	*LP_nmax_*(*μmol m^-2^ s^-1^*)	*LCP* (*μmol m^-2^ s^-1^*)	*LSP* (*μmol m^-2^ s^-1^*)	*R^2^*
W	0.04 ± 0.00^a^	0.65 ± 0.01^b^	5.67 ± 0.32^a^	77.61 ± 1.39^b^	750.52 ± 113.01^a^	0.998
R	0.03 ± 0.01^a^	0.73 ± 0.01^a^	4.45 ± 0.27^b^	90.61 ± 4.45^a^	650.51 ± 70.97^ab^	0.995
R3B1	0.05 ± 0.00^a^	0.31 ± 0.01^c^	4.52 ± 0.27^b^	34.38 ± 1.45^c^	543.50 ± 33.17^b^	0.995
R1B3	0.05 ± 0.00^a^	0.29 ± 0.01^d^	6.12 ± 0.16^a^	33.22 ± 1.90^c^	743.94 ± 70.32^a^	0.994

Different lowercase letters indicate significant differences between different treatments (*P* < 0.05). The same as below. Apparent Quantum Efficiency (*AQE*), Dark Respiration Rate (*R_d_*), Light Compensation Point (*LCP*), Light-saturated *P_n_* Maximum (*LP_nmax_*) and Light Saturation Point (*LSP*), coefficient of determination (*R*^2^). White: W, Red: R, R3B1 and R1B3: The ratio of photosynthetic photon flux density (PPFD) between red light and blue light is 3:1and 1:3.

**Figure 5:**
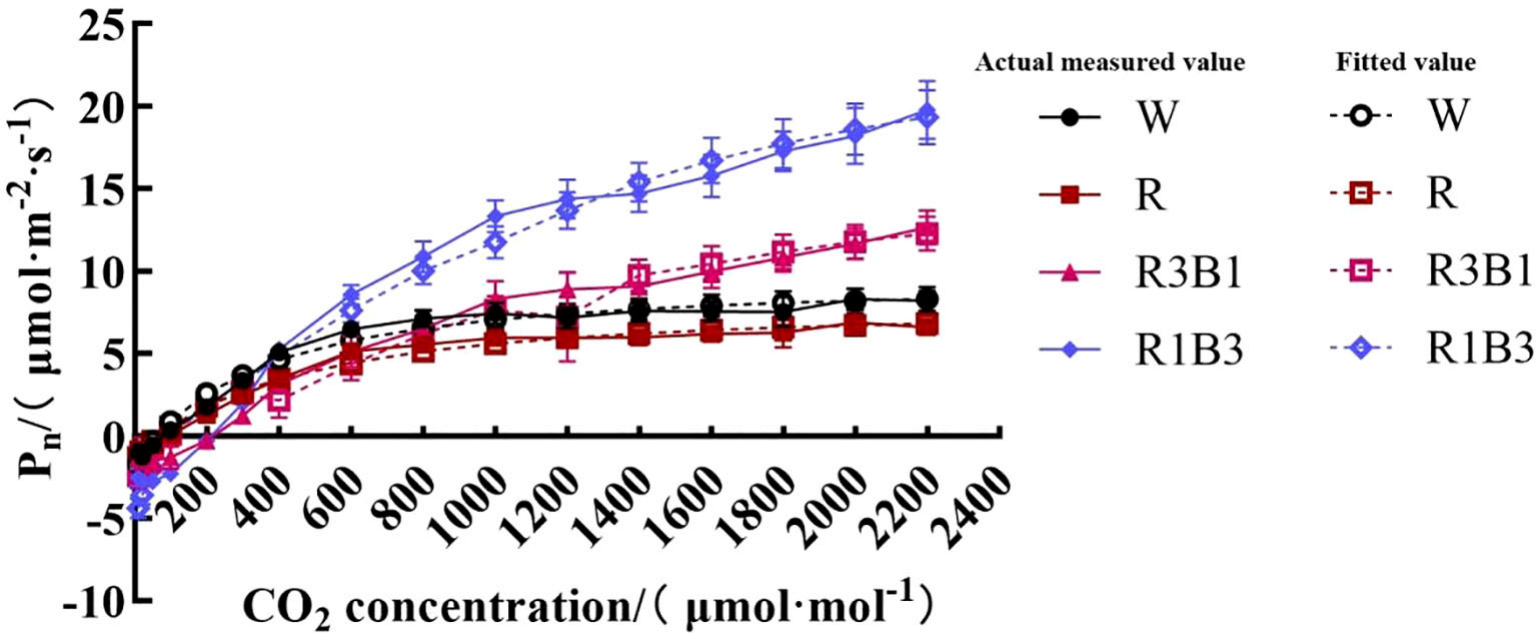
The response curves of *P_n_*-CO_2_ of *P.szechuanica* var. *tibetica* under different treatments. Of which the dashed lines represent fitted values, and the solid lines represent measured values. White: W, Red: R, R3B1 and R1B3: The ratio of photosynthetic photon flux density (PPFD) between red light and blue light is 3:1and 1:3.

The CO_2_ response curve reflected the law of *P*_n_ change of the *P. szechuanica* var. *tibetica* leaves with the change of intercellular CO_2_ concentration. As shown in **Figure 5**, the *P*_n_ of the leaves in different treatments increased rapidly when *C_i_* was in the range of 0 to 600 μmol·mol^-1^. When *C_i_* was within the range of 600 to 2200 μmol·mol^-1^, the *P*_n_ of the leaves treated with the W and R increased at a lower rate with the increase of *C*_i_ and tended to be stable, while the *P*_n_ of R3B1 and R1B3 increased with the increase of *C_i_*.

We calculated the *CSP*, *CCP*, maximum photosynthetic capacity (*CP*_max_) and photorespiration rate (*R*_p_) as per the CO_2_ response curve fitting formula (**Table 5**). The R^2^ values of all values models were higher than 0.9, which indicated that all models were highly efficient. The R1B3 showed higher average *CP*_max_, *CSP* and *CCP* values than the R, R3B1 and W treatments.

**Table 5:** Comparison of *P*_n-_*CO_2_* response parameters of *P. szechuanica* var. *tibetica* under different light quality.

Treatment	*CP_nmax_* (*μmol m^-2^ s^-1^*)	*α* (*μmol m^-2^ s^-1^*)	*R_p_* (*μmol m^-2^ s^-1^*)	*CSP* (*μmol m^-2^ s^-1^*)	*CCP* (*μmol m^-2^ s^-1^*)	*R^2^*
W	12.33 ± 1.00^c^	0.06 ± 0.00^b^	2.83 ± 0.11^c^	263.30 ± 7.08^c^	49.18 ± 0.38^b^	0.982
R	10.07 ± 0.83^c^	0.04 ± 0.00^b^	1.99 ± 0.00^c^	337.52 ± 26.18^b^	55.77 ± 0.47^b^	0.983
R3B1	22.38 ± 1.54^b^	0.29 ± 0.02^a^	13.98 ± 2.00^a^	131.42 ± 29.73^d^	50.03 ± 9.10^b^	0.923
R1B3	36.30 ± 3.87^a^	0.06 ± 0.01^b^	7.30 ± 1.31^b^	706.21 ± 4.78^a^	117.63 ± 6.40^a^	0.978

Different lowercase letters indicate significant differences between different treatments (*P* < 0.05). The same as below. The letters in the table represent: CO_2_-saturated maximum *P_n_* (*CP_nmax_*), Carboxylation Efficiency (*α*), Photorespiration Rate (*R_p_*), CO_2_ Saturation Point (*CSP*) and CO_2_ Compensation Point (*CCP*). White: W, Red: R, R3B1 and R1B3: The ratio of photosynthetic photon flux density (PPFD) between red light and blue light is 3:1and 1:3.

### 3.4 Impact of different light treatments on photosynthetic pigments and products

**Figure 6:**
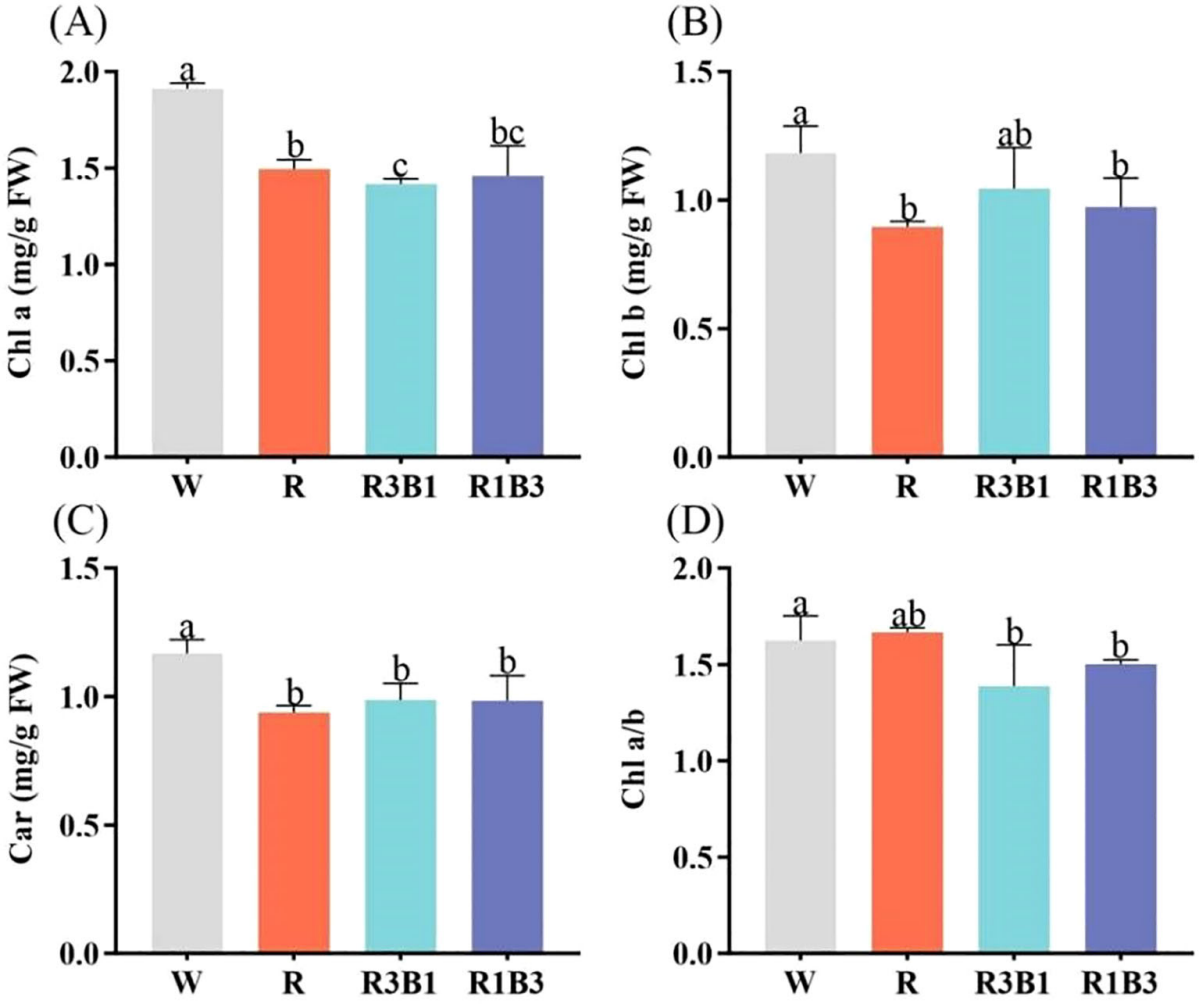
Effect of different light quality treatments on pigment contents. **(A)** Chl a, **(B)** Chl b, **(C)** Car, and **(D)** Chl a/b. Different lowercase letters indicate significant differences between different treatments (*P* < 0.05). White: W, Red: R, R3B1 and R1B3: The ratio of photosynthetic photon flux density (PPFD) between red light and blue light is 3:1and 1:3.

The content of Chl a, Chl b and Car in leaves was affected by light quality (**Figure 6**). Compared with W, R, R3B1 and R1B3 treatments decreased the content of Chl a, Chl b and Car (*P* < 0.05; **Figures 6A, B, C**). There were no significant difference in contents of Car and Chl a/b under the light qualities of R, R3B1 and R1B3 (*P*>0.05; **Figures 6C, D**).

**Figure 7:**
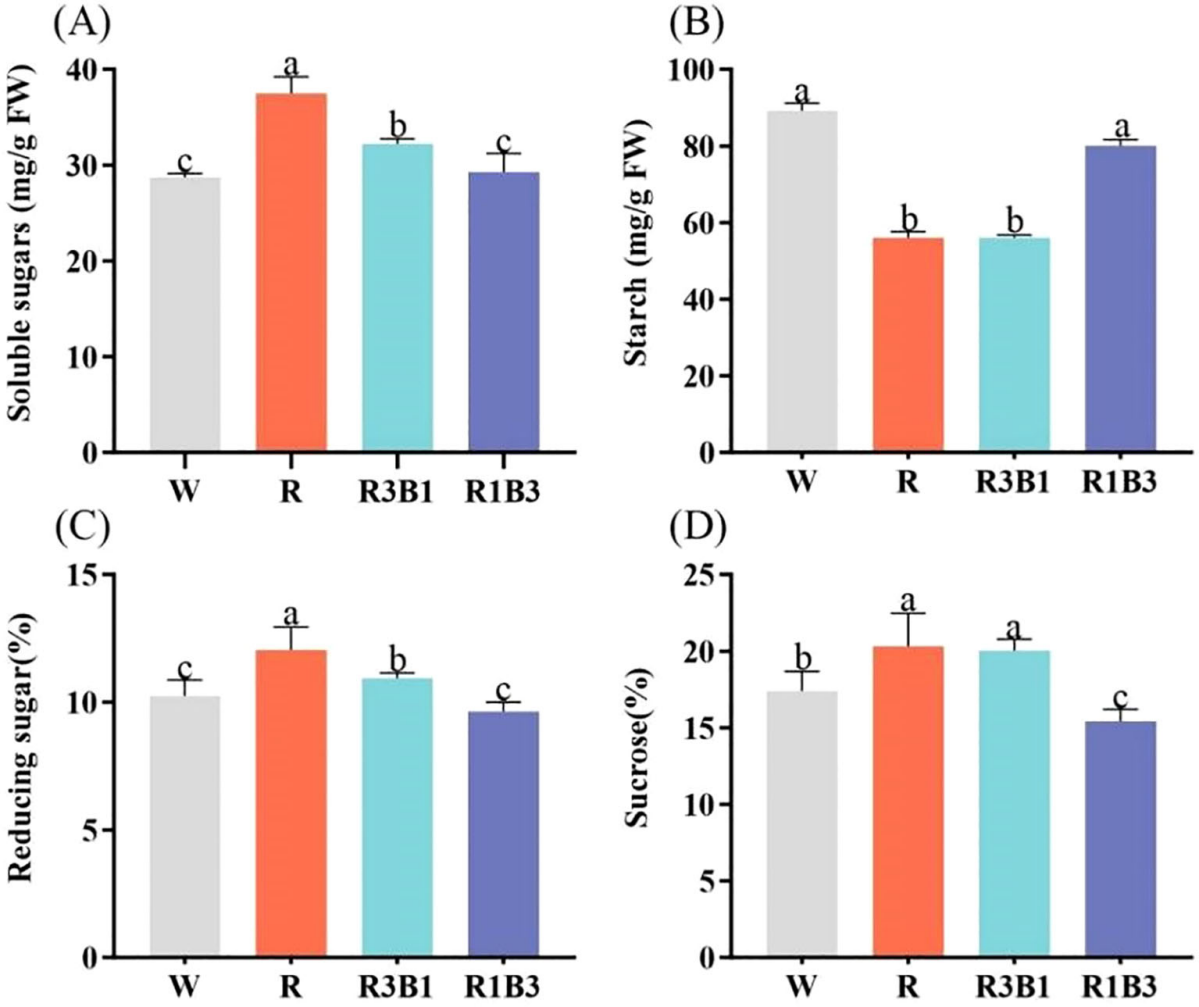
Effect of different treatments on soluble sugars **(A)**, starch **(B)**, reducing sugar **(C)** and sucrose **(D)** content. Different lowercase letters indicate significant differences between different treatments (*P* < 0.05). White: W, Red: R, R3B1 and R1B3: The ratio of photosynthetic photon flux density (PPFD) between red light and blue light is 3:1and 1:3.

The effects of different light qualities on the photosynthetic products of *P. szechuanica* var. *tibetica* are presented in **Figure 7**. The highest contents of total soluble sugar, reducing sugar and sucrose were observed in the R treatment, at 37.50 mg/g, 12.05% and 20.30%, respectively-representing increases of 30.53%, 22.07% and 16.73% compared to W treatment (28.73 mg/g, 10.24% and 17.39%) (**Figures 7A, C, D**). The R3B1 treatment followed in magnitude (**Figures 7A, C, D**). Under R and R3B1 treatments, the plants showed the lowest content of starch (56.09 mg/g and 56.09 mg/g), which was significantly decreased by 33.07 and 37.09% (**Figure 7B**).

### 3.5 Impact of different light treatments on the content and activity of photosynthetic enzymes

**Figure 8:**
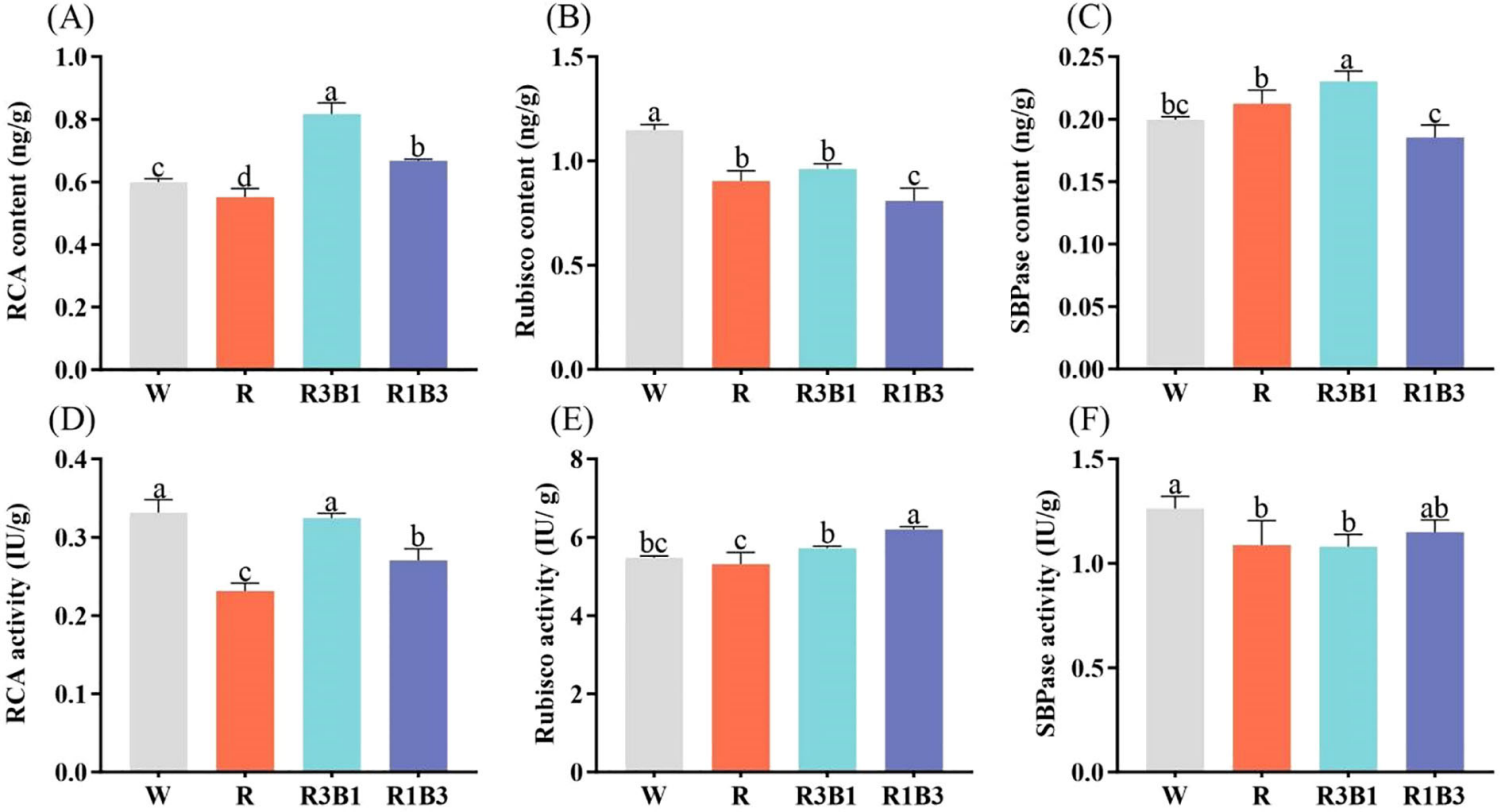
Index of photosynthetic enzyme contents and activities after light treatment. **(A)** RCA content of *P. szechuanica* var. *tibetica* after light treatments; **(B)** Rubisco content of *P. szechuanica* var. *tibetica* after light treatments; **(C)** SBPase content of *P. szechuanica* var. *tibetica* after light treatments; **(D)** RCA activity of *P. szechuanica* var. *tibetica* after light treatments; **(E)** Rubisco activity of *P. szechuanica* var. *tibetica* after light treatments; **(C)** SBPase activity of *P. szechuanica* var. *tibetica* after light treatments. White: W, Red: R, R3B1 and R1B3: The ratio of photosynthetic photon flux density (PPFD) between red light and blue light is 3:1and 1:3. Different lowercase letters indicate significant differences between different treatments (*P* < 0.05).

To investigate the effects of red and blue light on the content and activity of photosynthesis-related enzymes, the seedlings were exposed to different light qualities for 40 days. The results showed that the content and activity of RCA, Rubisco, and SBPase were different significantly among the treatments (**Figure 8**). The light qualities significantly decreased the Rubisco content compared to the CK treatment, but the R and R3B1 treatments did not show a significant difference (**Figure 8B**). The highest RCA content was in the R3B1 treatment (36.67% higher than that in the W treatment), followed by the R1B3 treatment (0.67ng/g). The lowest RCA content was in the R treatment (0.55 ng/g). As indicated in **Figure 8C**, the highest and lowest value for SBPase content were obtained in R3B1 and R1B3 treatment, respectively.

Significant differences were observed in the effects of various light treatments on the activity of RCA, Rubisco, and SBPase. Noteworthy is the observation that under R treatment, RCA, Rubisco, and SBPase activities were 30.3%, 3.1% and 13.49% lower, respectively, than CK treatment (**Figures 8D-F**). Moreover, the Rubisco activity under R1B3 and R3B1 treatments showed significantly higher rates compared to the CK treatment.

## 4 Discussion

The light response curve depicts the trend of photosynthetic rate changes with photosynthetically active radiation, and serves as a key indicator of plant photosynthetic capacity (Li et al., 2023). *LP_nmax_* denotes the maximum photosynthetic potential of plant leaves (Wu et al., 2019). The two parameters, *R_d_*, and *LCP*, all reflect a plant’s low-light utilization capacity (Liang et al., 2023; Shang et al., 2020). The CO_2_ response curve reflects the plant’s CO_2_ utilization capacity (Liu et al., 2015). In this study, exposure to monochromatic red light resulted in an increased light compensation point (*LCP*) and higher dark respiration rate (*R_d_*), indicating a reduced ability to utilize low light. In contrast, the introduction of blue light, particularly under the R1B3 treatment, significantly lowered the *LCP*. Concurrently, key photosynthetic parameters, including the net photosynthetic rate (*P_n_*), light-saturation maximum net photosynthetic rate (*LP_nmax_*), and CO_2_-saturation maximum net photosynthetic rate (*CP_nmax_*_)_, were markedly enhanced under R1B3, collectively leading to superior photosynthetic performance in the poplar plants. The structure of the leaves is closely associated with its various functions (Song et al., 2021). Stomata serve as crucial pathways for water cycling and CO_2_ exchange in ecosystems, being regulated by both physiological and environmental factors (Wang et al., 2008). From the leaf anatomical structure perspective, the number of stomata on the upper epidermis significantly increased under the R1B3 treatment in this study. Although the number of stomata on the lower epidermis significantly decreased, this represents a quantitative change. In terms of (stomatal quality) stomatal conductance (*G_s_*), stomatal conductance significantly increased under this treatment, reaching the same level as the control (W). The reduction in the number of stomata on the lower epidermis may be related to the thickening of the lower epidermis and the tightening of its tissue structure. Research has demonstrated that blue light can promote stomatal opening (Horrer et al., 2016). enhancing leaf gas exchange capacity can be achieved by increasing the thickness of the lower epidermis (Wang et al., 2022). Similar to this study, R1B3 enhances photosynthetic performance by increasing stomatal conductance (stomatal quality).

Leaves capture light energy via photosynthetic pigments to drive photosynthesis, producing initial organic products; thus, their carbohydrate synthesis and metabolism are also regulated by different light qualities (Zhang and Wang, 2021). Under light conditions, leaf chloroplasts utilize photochemical energy to fix CO_2_: part of the photosynthetic products is exported from chloroplasts to meet immediate respiratory demands or for sucrose export, while another part is temporarily stored as starch to support nocturnal respiration and periods of carbon surplus (MacNeill et al., 2017). In this study, the contents of soluble sugar, reducing sugar, and sucrose in the R and R3B1 treatments were significantly higher than those in the W and R1B3 treatments, whereas starch content was significantly lower. This indicates that red light promotes the synthesis of soluble sugar, reducing sugar, and sucrose, while blue light promotes starch synthesis. This phenomenon may arise because red light enhances cell elongation and strengthens respiration, thereby increasing energy demand and reducing surplus energy substances, which in turn decreases starch synthesis; blue light exhibits the opposite effect.

Chlorophyll and carotenoids are the primary photosynthetic pigments in plants, existing as protein complexes in the thylakoid membranes of chloroplasts. They participate in the energy conversion processes of photosynthesis by absorbing and transferring light energy, and changes in their content are associated with plant photosynthetic performance (Zhang et al., 2024). In this study, all three treatment groups inhibited the synthesis of photosynthetic pigments; however, treatments containing blue light showed a modest improvement compared to those without blue light.

Rubisco activity is inhibited by sugar phosphate derivatives. RCA utilizes energy from ATP hydrolysis to promote the release of these derivatives, which are then dephosphorylated by phosphatases upon dissociation from the catalytic site, thereby preventing their rebinding to Rubisco (Orr and Robijns, 2023). SBPase participates in the regeneration of ribulose-1,5-bisphosphate (RuBP) and acts as a limiting factor for carbon fixation. Previous studies have shown that red light enhances the activities of Rubisco, SBPase, and RCA in tomato seedlings (Li and Liu, 2021). However, in this study, red light inhibited the activities of all three enzymes, whereas blue light supplementation enhanced RCA and Rubisco activities. Rubisco activity increased with higher blue light proportions, with significantly higher activity under the R1B3 treatment compared to the W treatment. Similar results were reported in rice under different light quality treatments, where blue light significantly increased Rubisco activity while red light suppressed it (Ren and Liu, 2023).

Studies have demonstrated that single red light application typically induces the “red light effect” in plants, resulting in photosynthetic disorders, whereas blue light can mitigate this effect (Di et al., 2020; Miao et al., 2019; Wang et al., 2015). These findings align with previous studies demonstrating that combined red-blue light promotes superior plant growth compared to monochromatic treatments (Hernández et al., 2016; Hogewoning et al., 2010). *Ginkgo biloba* showed optimal development under a 1:1 red: blue ratio (Wang and Zhang, 2024). Cucumber (*Cucumis sativus*) achieved higher net photosynthetic rate, plant height, and leaf area under 9:1 red: blue light, leading to improved subsequent growth (Wang et al., 2024). *Morus alba* exhibited reduced adverse effects under mixed red-blue light compared to single-spectrum treatments (Hu and Dai, 2019).

Generally speaking, as the altitude gradient increases, the light intensity rises and the short-wave radiation strengthens (Pan et al., 2009). Plants at different altitudes can adapt to the light environment through morphological changes (Wang, 1989; Castro et al., 2000). Under high blue light conditions (R1B3) in this study, *P.szechuanica* var. *tibetica* leaves became shorter and thicker, while plants developed a compact, stunted growth habit. However, stomatal conductance increased and photosynthetic performance improved. This aligns with the adaptive response mechanism of plants in high-altitude regions to elevated blue light ratios, consistent with previous findings. Phenotype studies of *Populus cathayana* leaves across different populations revealed that small-leaf morphology predominates at high elevations (Cao et al., 2021). In *Salix sclerophylla*, leaf thickness increased with altitude (Guo et al., 2019). Studies on *Platycladus orientalis*, *Prunus davidiana*, and *Tamarix austromongolica* revealed that higher elevations increased photosynthetic rate (*P_n_*) (Liu et al., 2021). Blue light is a crucial light quality for plant development. Exposure to blue light stimulates the expression of genes such as *CRY*. Research indicates that *CRY1* is activated by blue light stimulation, influencing genes involved in plant hormone signal transduction through site competition, thereby altering endogenous hormone levels (Feng et al., 2017; Ludwig-Müller et al., 2009). This interplay between light signals and endogenous hormone signaling-a collaborative process involving light quality, genes, and hormones-significantly impacts plant photosynthetic traits, stomatal opening/closing, and growth development (Yan and Rong, 2017). It may explain the underlying mechanism behind the response observed in the *P.szechuanica* var. *tibetica*.

This study elucidates the response mechanisms of *P szechuanica* var. *tibetica*, a tree species native to high-altitude regions, to prolonged exposure to short-wavelength-enriched light. The findings provide a theoretical basis for investigating altitude species divergence and for the informed introduction of tree species to high-altitude areas. This research, however, has certain limitations. These include the absence of a pure blue light treatment and the unexplored molecular mechanisms underlying the phenotype and physiological changes induced by the R1B3 light regime. Future work will incorporate pure blue light and other mixed light qualities, employ transcript sequencing to identify key regulatory genes under different light conditions, and conduct functional validation experiments to deepen our understanding of how red and blue light govern the growth and development of *Populus szechuanica* var. *tibetica*.

## 5 Conclusions

In summary, this study demonstrates that *Populus szechuanica* var. *tibetica* have evolved unique response mechanisms to adapt to the altered light quality (dominated by blue light) in high-altitude regions. Monochromatic red light induces plant elongation and weakens photosynthetic performance, whereas under specific red-blue mixed light (R1B3) conditions, plants exhibit a compact morphology with smaller, thicker leaves, enhanced stomatal conductance, and increased Rubisco activity, thereby boosting photosynthetic capacity. This study reveals the physiological adaptation mechanisms of tree species in high-altitude regions to light conditions with high blue light proportions. It provides actionable strategies for investigating differences in photosynthetic mechanisms among tree species across varying altitudes and for introducing tree species from high-altitude regions.

## Data Availability

The raw data supporting the conclusions of this article will be made available by the authors, without undue reservation.
